# Injectable thermo-responsive hydrogel scaffold for enhanced delivery of second-generation induced neural stem cells for treatment of glioblastoma

**DOI:** 10.21203/rs.3.rs-8398602/v1

**Published:** 2026-01-16

**Authors:** Kayla M. Maue, N our Caroline Dau, Jasmine L. King, Morrent Thang, Shawn D. Hingtgen, Soumya Rahima Benhabbour

**Affiliations:** University of North Carolina at Chapel Hill; University of North Carolina at Chapel Hill; University of North Carolina at Chapel Hill; University of North Carolina at Chapel Hill; University of North Carolina at Chapel Hill; University of North Carolina at Chapel Hill

**Keywords:** TRAIL, neural stem cell, chitosan, glioblastoma, injectable hydrogel

## Abstract

Glioblastoma (GBM) remains a highly aggressive brain tumor with poor prognosis despite surgical resection and standard chemoradiation. Induced neural stem cell (iNSC)-based therapies offer a promising strategy owing to their inherent tumor-homing ability and capacity to deliver therapeutics selectively to the tumor site; however, their poor retention within the tumor resection cavity limits clinical potential. Herein, we evaluated second-generation TRAIL-secreting iNSCs (hiNeuroS) in combination with a biodegradable, thermo-responsive chitosan hydrogel for localized and sustained delivery of high densities of hiNeuroS. Direct comparisons with first-generation iNSCs (hiNSCs) demonstrated that hiNeuroS achieved faster and more extensive tumor cell kill across multiple tumor-to-therapeutic cell ratios in both U87 and patient-derived GBM8 models. Injectable chitosan scaffolds supported stem cell densities up to 2 × 10^7^ cells/mL, maintained > 90% viability, preserved scaffold microstructure, and maintained rapid gelation at physiological conditions. Gravimetric analysis revealed stable 30-day mass change profiles, with minimal net mass loss indicating preserved scaffold integrity. Moreover, encapsulated hiNeuroS retained their migratory capacity and sustained TRAIL secretion, inducing significant tumor cell death in both GBM models. Collectively, these findings demonstrate that hiNeuroS maintain functional potency in GBM and are compatible with scaffold-based delivery. This work provides a foundation for future in vivo studies to assess scaffold-mediated retention, persistence, and therapeutic efficacy in an established GBM mouse model, supporting the development of an injectable hydrogel platform for next-generation cell-based therapies for treatment of GBM and other malignant tumors.

## Introduction

Glioblastoma (GBM) remains the most lethal primary brain malignancy, with over 12,000 new cases diagnosed annually in the US and a median survival of only 12–15 months despite maximal surgical resection and standard chemoradiation [1–3]. Limited progress over the past two decades reflects the biological barriers inherent to GBM, including diffuse infiltrative invasion that prevents complete tumor resection, extensive tumoral heterogeneity, a highly immunosuppressive tumor microenvironment (TME), and the restrictive blood-brain barrier (BBB) that limits systemic drug delivery [4–6]. As a result, recurrence is nearly universal and long-term survival is rare, highlighting the urgent need for alternative therapeutic strategies capable of addressing residual infiltrative cancer cells following resection [7].

Currently, there is no established standard of care for recurrent GBM, with treatment options remaining limited. Conventional chemotherapies and immunotherapies have shown minimal clinical benefit in recurrent GBM due to limited tumor penetration, rapid development of resistance, and failure to overcome the immunosuppressive nature of GBM [8, 9]. These challenges have fueled interest in therapeutic approaches that pair tumor-selective targeting with sustained, localized delivery. Cell-based therapies are particularly attractive due to cell signaling pathways capable of responding to complex microenvironments, migrating toward pathological sites, and delivering therapeutic payloads with high specificity [10]. Neural stem cells (NSCs) are promising in this regard due to their intrinsic tumor-tropic migration [11]. Induced neural stem cells (iNSCs), generated by direct reprogramming of autologous somatic cells, further enable personalized therapy and genetic engineering for targeted payload delivery [12–14]. One molecule of interest, tumor necrosis factor related apoptosis inducing ligand (TRAIL) has been shown to selectively induce apoptosis in cancer cells via death receptors 4 and 5, while sparing healthy tissue [15, 16]. Our laboratory previously investigated delivery of first-generation TRAIL-secreting iNSCs (hiNSCs), produced from a patient’s fibroblasts in adherent culture, that demonstrated tumor-selective cytotoxicity and therapeutic efficacy in preclinical glioma and brain metastases models [17–19]. Building on this platform, second-generation induced NSCs (hiNeuroS) were developed to enhance proliferative capacity, migratory potential, and stem cell-like properties with a spheroidal morphology, resulting in robust tumor tropism and prolonged persistence [20]. These combined cellular improvements translated to enhanced therapeutic efficacy in breast cancer brain metastasis models, achieving a 40% increase in median survival compared to hiNSCs [20]. Notably, in vivo studies with hiNeuroS combined with radiation demonstrated significant improvements (36.6%) in median survival in non-small cell lung cancer (NSCLC) brain metastasis models compared to controls [21]. Although promising, translation of these hiNeuroS advantages to recurrent GBM, which presents a distinct and more complex tumor microenvironment, remains unknown and is worth investigating.

Key barriers to clinical translation of cell-based therapies include their poor retention and survival once injected due to rapid clearance, and the BBB that further restricts therapeutic access [22, 23]. Consistent with these limitations, a murine GBM resection model demonstrated that approximately 19% of directly injected therapeutic stem cells persisted after 7 days, with complete clearance by day 10 [24]. Localized delivery strategies have therefore gained attention as a way to improve therapeutic persistence at the resection site. Gliadel^®^ wafers established the clinical precedent that biomaterial-based agents can be safely placed within the resection cavity to bypass the BBB and provide sustained local therapy [25]. Building on this concept, polymer-based scaffolds are now being explored for cell delivery, as they can provide physical retention, structural support, and a microenvironment that enhances cell survival, persistence, and function.

While electrospun and 3D-printed scaffolds have shown promise for the delivery of cellular therapies [26, 27], they have fixed geometries which can limit conformity to the irregular and patient-specific anatomy of a GBM resection cavity. In a clinical setting, gaps between the scaffold and the resection margin may reduce exposure of residual infiltrative tumor cells to the therapeutic payload and potentially limit treatment efficacy. In contrast, injectable, thermogelling hydrogels can fill the cavity in situ and be delivered during the same surgical procedure, reducing the need for additional implantation steps [28, 29]. Critically, injectable hydrogels enable surgical flexibility by allowing clinicians to tailor injection volume, dosing, and cavity conformity to individual patient anatomy during the procedure itself.

To address these needs, our laboratory developed a thermoresponsive chitosan (CS)-based hydrogel system that transitions from liquid to gel at physiological temperature, forming a supportive three-dimensional matrix for stem cell encapsulation. Chitosan offers several advantages as a biomaterial platform, including biocompatibility, biodegradability, and inherent antimicrobial properties [30–32]. The biodegradable nature of the scaffold is particularly advantageous clinically, as it eliminates the need for a second surgical procedure to remove an inert implant. The platform’s shear-thinning and self-healing behavior supports smooth injection and uniform cell distribution, offering a practical and adaptable approach for localized cell delivery [33, 34]. Originally developed for bone regeneration [35, 36], this platform was later adapted for delivery of iNSCs for GBM treatment [37, 38]. Prior work with first-generation iNSCs demonstrated that the CS scaffold promoted viability of hiNSCs, enabled TRAIL secretion, and improved median survival in murine GBM resection models with favorable safety and persistence profiles [37, 38]. However, practical cell seeding density was limited to 1 × 10^7^ cells/mL due to viscosity constraints, and prior studies primarily used U87 glioma cells, which do not fully represent GBM heterogeneity [39, 40]. The current study addressed these limitations by evaluating hiNeuroS at higher cell densities and expanding tumor model representation by incorporating patient-derived GBM cells.

Herein we investigated the prolonged delivery of second-generation TRAIL-secreting hiNeuroS with an injectable chitosan scaffold capable of accommodating high cell densities to advance the translational potential of iNSC-based therapy for GBM. We first performed a direct head-to-head comparison of hiNSCs and hiNeuroS to evaluate therapeutic potency in both U87 and patient-derived GBM8 cells. We then characterized hiNeuroS-loaded CS hydrogels seeded at densities up to 2 × 10^7^ cells/mL by assessing mechanical and rheological properties, scaffold stability, stem cell viability, TRAIL secretion, and tumor-killing efficacy in vitro. hiNeuroS demonstrated superior cytotoxicity across multiple tumor-to-therapeutic cell ratios, while the CS scaffold supported high cell density encapsulation, maintained > 90% cell viability, preserved microstructure, elicited rapid gelation kinetics, and enabled sustained TRAIL secretion that resulted in potent tumor cell death.

To our knowledge, this work provides the first direct comparison of first- and second-generation iNSCs specifically in the GBM context, as well as the first evaluation of hiNeuroS performance when delivered within a hydrogel scaffold. By integrating an improved cellular therapeutic with a clinically viable, injectable biomaterial platform, this study advances a next-generation strategy for improved GBM treatment and lays essential groundwork for translational development.

## Materials and Methods

### Materials

Chitosan (CS) powder (MW: 310–375 kDa, 75% deacetylated, 800–2000 CPS (C = 1%, 1% acetic acid), Lot #: BCCC6283), β-glycerophosphate (BGP, Lot #: SLCM7561), and hydroxyethyl cellulose (HEC, MW: 90,000 Da, Lot #: SLCM7561) were purchased from Sigma-Aldrich (St. Louis, MO, USA). 1 mL Luer-Lock syringes and BD PrecisionGlide^™^ 18 G × 0.5” hypodermic blunt tip needles were purchased from Becton Dickinson (Franklin Lakes, NJ, USA). Luer-Lock connectors were purchased from Baxter (Deerfield, IL, USA). NORM-JECT All-Plastic 1 mL syringes were obtained from Grainger (Lake Forest, IL, USA).

The LIVE/DEAD Viability/Cytotoxicity Kit containing Calcein AM and Ethidium Homodimer-1 reagents, 8-Well Borosilicate Glass Chambered Coverglass, and D-luciferin potassium salt were purchased from Thermo Fisher Scientific (Waltham, MA, USA). Human TRAIL ELISA Kits (CD253) were purchased from Abcam (Cambridge, United Kingdom). Falcon cell strainers (100 μm mesh size) were purchased from Fisher Scientific (Pittsburgh, PA, USA).

Standard cell culture medium consisted of Dulbecco’s Modified Eagle Medium (DMEM; Gibco, Detroit, MI, USA) supplemented with 10% fetal bovine serum (FBS; Gibco), 1% penicillin/ streptomycin (P/S; Gibco), and 4 mM L-glutamine (Gibco). Dulbecco’s phosphate-buffered saline (DPBS; Gibco) and Neurobasal^™^ medium (Gibco) were obtained from the University of North Carolina Tissue Culture Facility (Chapel Hill, NC, USA). STEMdiff^™^ Neural Induction Medium and Accutase^™^ Cell Detachment Solution were purchased from STEMCELL Technologies (Cambridge, MA, USA). Doxycycline powder was purchased from Takara Bio (San Jose, CA, USA). Additional supplements included L-glutamine (200 mM, 100 ×; Gibco), B27 Supplement (50 ×; Thermo Fisher), N2 Supplement (100 ×; Thermo Fisher), Antibiotic-antimycotic (Anti-anti; 1%, Gibco) and heparin (2%; STEMCELL Technologies). Recombinant human epidermal growth factor (EGF) and fibroblast growth factor (FGF) were purchased from Fujifilm (Tokyo, Japan). ReNcell NSC Maintenance Medium was obtained from Sigma-Aldrich (St. Louis, MO, USA).

### Cell lines

U87-MG tumor cells were obtained from the American Type Culture Collection (ATCC). GBM8 cells were gifted by H. Wakimoto (Massachusetts General Hospital). hTERT-immortalized normal human fibroblasts (NHF-1) were obtained from W. Kauffmann (University of North Carolina School of Medicine). U87-MG human glioblastoma cells were cultured in standard cell culture medium and maintained at 37°C in a humidified 5% CO_2_ incubator. GBM8 patient-derived glioblastoma stem-like cells were cultured in filtered EF medium consisting of Neurobasal^™^ medium (500 mL; Gibco) supplemented with 3 mM L-glutamine, 10 mL B27 supplement, 2.5 mL N2 supplement, 2 mg/mL heparin, 1% P/S, 1% anti-anti, and 20 ng/mL each of recombinant human EGF and FGF. Cells were maintained at 37°C in 5% CO_2_ and dissociated into single-cell suspensions using Accutase prior to use.

### Lentiviral vectors

Lentiviral vectors were kindly provided by the Duke University Viral Vector Core (Durham, NC, USA). The following constructs were used: (1) reverse tetracycline-controlled transactivator (rtTA), (2) doxycycline-inducible SOX2 (SOX2), (3) mCherry–Firefly Luciferase (mCh-Fluc), (4) GFP–Firefly Luciferase (GFP-Fluc), and (5) GFP fused to a secretable TRAIL variant (GFP-TRAIL). The mCh-Fluc vector was used to transduce U87 and GBM8 cells for longitudinal monitoring of fluorescence and bioluminescence during experiments. Transduced cell lines are denoted as U87-mCh-Fluc and GBM8-mCh-Fluc, respectively.

### Induced neural stem cell generation

First-generation iNSCs (hiNSC-TRAIL) were generated following an established protocol [18]. Briefly, 2 × 10^6^ NHF-1 human fibroblasts were transduced with either LV-GFP-sTRAIL or LV-mCherry-FLuc, plated in T-175 flasks and cultured in standard cell culture medium (Day 1). On Day 2, the medium was replaced with Neural Induction Medium containing 2 μg/mL doxycycline (NIM + doxy; also referred to as transdifferentiation (TD) medium). The TD medium was replaced every other day for 4 days. On Day 6, hiNSC-TRAIL cells were collected, dissociated using Accutase and filtered through a 100 μm cell strainer.

Second-generation neurospheres engineered to secrete TRAIL (hiNeuroS-TRAIL) were generated using an established protocol [20]. Briefly, following transduction of NHF-1 human fibroblasts, 1 × 10^5^ cells were seeded in six-well plates with supplemented 10% FBS DMEM media overnight. Starting from day 2 after the initial transduction, cells were cultured in ReNcell NSC maintenance medium supplemented with EGF and FGF (20 ng/mL) and doxycycline (2 μg/mL). Spheres were collected from media by centrifugation and seeded in well plates precoated with laminin (10 mg/mL in PBS; Sigma). Nonadherent cells were collected after culturing overnight and reseeded in laminin-coated plates. This floating spheres collection and reattachment process was repeated three times before spheres were fully expanded for use. hiNeuroS-TRAIL cells were maintained in ReNcell NSC Maintenance Medium supplemented with doxycycline, recombinant human EGF and FGF. Cells were incubated at 37°C in 5% CO_2_ and passaged by centrifugation. Prior to use, cells were dissociated into single-cell suspensions using Accutase.

### In vitro coculture assay

Human U87-MG-FLuc and GBM8-FLuc brain tumor cells were cocultured directly with first- and second-generation iNSCs at various tumor cell to stem cell ratios (10:1, 5:1, 1:1, 1:5) and therapeutic efficacy assessed using bioluminescence imaging (BLI). To perform in vitro coculture assays, U87-MG-FLuc and GBM8-FLuc cells were each cocultured with either first- or second-generation iNSCs in 96-well plates. Tumor cells alone in standard media, either DMEM or EF appropriate to tumor cell line, served as experimental controls. Various hiNSC-TRAIL and hiNeuroS-TRAIL cell counts were prepared via centrifugation, dissociation with accutase, and neutralization with PBS. After resuspension in PBS, 10 μL of cells were combined with 10 μL of Trypan blue and placed in counting chamber slides using Invitrogen Countess Cell Counter. Desired cell counts for each tumor cell line and therapeutic cell line were calculated and resuspended together in standard media for use. 150 μL of this coculture solution was added to each well, and this was repeated for each tumor to therapeutic cell ratio. At 24 h, 48 h and 72 h, termination assays were conducted using bioluminescent imaging (BLI) to quantify tumor signals by adding 150 μL 10% firefly-luciferin in PBS (prepared from 15 mg/mL stock solution) to each well of the treated 96-well plate. After 15 min of incubation, BLI images were captured using a Spectral Instruments AMI instrument with an exposure of 5s.

### Preparation of hydrogel formulation

A 3% w/v chitosan (CS) stock solution was prepared as previously reported by stirring chitosan powder in 0.1 M acetic acid in deionized water at room temperature for 48 h and sterilized under UV light [35]. 1 M β-glycerophosphate (BGP) stock solution was prepared by dissolving BGP in deionized water, and hydroxyethyl cellulose (HEC) stock solution (25 mg/mL) was prepared by dissolving HEC in serum free DMEM media. BGP and HEC stock solutions were sterile filtered before use. The injectable thermogelling hydrogel scaffolds were formed using a three-component stepwise mixing system under aseptic conditions. The three separate components consisted of: (1) CS, (2) BGP, (3) HEC, with or without cells, in DMEM media. In brief, the first 2% w/v CS and BGP gelling components were combined and homogenously mixed using a Luer Lock connecter. The third component was transferred into the CS/BGP solution and mixed thoroughly to create a pre-hydrogel mixture, followed by incubation at 37°C for 30 min to form a hydrogel.

### Rheological analysis

Gelation times of CS-based hydrogels were determined using a rotational rheometer, DHR-3 (TA Instruments, New Castle, DE, USA) after preparation of the pre-hydrogel solution as previously described [36, 37]. Rheology measurements were obtained using an 8 mm diameter sandblasted parallel plate, controlled at 37°C by a Peltier Plate heating system. Samples (50 μL) were injected in the center of the lower plate and the upper plate was lowered to a 750 μm gap distance. The linear viscoelastic region (LVR) for the CS hydrogel was generated by running an oscillatory strain sweep from strain amplitudes 0.01% – 1000%. Optimization of strain amplitude (0.01%, 0.05%, and 0.1%) and angular frequency (10, 15, and 20 rad/s) showed that optimal results within the LVR of the hydrogel were obtained with 0.05% strain amplitude and angular frequency of 20 rad/s. Dynamic time sweeps were thus performed at 0.05% strain with an angular frequency of 20 rad/s for all rheological measurements.

### Scanning electron microscopy (SEM)

Hydrogels were prepared as previously described with a cell loading density of 5 × 10^6^ to 2 × 10^7^ iNSCs/mL of hydrogel (200 μL/mold, n = 3) and incubated at 37°C/5% CO_2_ in supplemented ReNcell for 24 h or 72 h. The hydrogel scaffolds were collected, flash frozen with liquid nitrogen, and lyophilized for 24 h. Lyophilized samples were bisected with surface cross section mounted upwards on 13 mm diameter aluminum stubs using carbon adhesive tabs before sputter coating with 8 nm of a 60:40 gold/palladium alloy using a Cressington 208HR Sputter Coater (Ted Pella Inc, Redding CA). Images were obtained using a Zeiss Supra 25 FESEM operating at 5 kV using an SE2 detector, 20 μm aperture, and approximate working distances of 5 to 8 mm (Carl Zeiss Microscopy, LLC, Peabody, MA).

### In vitro mass change study

The hydrogels were prepared as previously described using 1 mL Luer Lock syringe. Briefly, the pre-hydrogel mixture was injected into a NORM-JECT syringe mold using an 18 G needle (200 μL /mold, n = 6) and incubated at 37°C for 30 min. The hydrogel samples were subsequently transferred from the molds and seeded directly into a 12-well plate and cultured in supplemented ReNcell media, with media changes every two days. On days 0, 3, 7, 14, and 30, the hydrogel samples were collected, lyophilized for 24 h using FreeZone 4.5 Liter Freeze Dryer (Labconco, Kansas City, MO, USA), and weighed to determine mass changes using the following equation:

$$Net\;Mass\;Change\;(\%)=\frac{\left(Wf\;-\;Wi\right)}{Wi}\times 100$$

Where Wi is initial dry weight at day 0 and Wf is the final dry weight at given time point, for each respective formulation. Hydrated weights were also collected prior to lyophilization at each timepoint for insight into the scaffold’s water content and swelling capabilities.

### Cell viability studies

hiNeuroS were encapsulated in a 2% (w/v) chitosan solution at densities ranging from 5 × 10^6^ cells/mL to 2 × 10^7^ cells/mL hydrogel. 50 μL of the pre-hydrogel solution was seeded into 8-well plates (n = 4) and incubated at 37°C for 30 minutes to achieve complete gelation. 500 μL of ReNcell media, complete with Doxycycline and EGF + FGF growth factors, was added to each well. Scaffolds were incubated for 24 h and 72 h before cell viability was assessed using LIVE/DEAD^®^ Viability/Cytotoxicity kit (Invitrogen, Waltham, MA, USA). The fluorescent images were captured at 10 × magnification using a laser scanning confocal microscope (Zeiss LSM780, Jena, Germany). Percent cell viability was calculated using Image J cell counter analysis.

### In vitro TRAIL release quantification

CS hydrogels were prepared as previously described with a cell loading density of 5 × 10^6^ to 2 × 10^7^ iNSC/mL of hydrogel (100 μL/mold, n = 6) and incubated at 37°C/5% CO_2_. Hydrogel samples were prepared in individual syringe molds and subsequently seeded in a 12-well plate and cultured in complete ReNcell media for 24 h and 72 h. Following incubation, 1 mL aliquots of TRAIL conditioned media were collected and centrifuged at 1000 rpm for 10 minutes to remove any cells and debris and media was frozen at −80°C until use. To measure the amount of TRAIL released from the cell-laden hydrogel scaffolds, an in vitro Human TRAIL Enzyme-Linked Immunosorbent Assay (ELISA) was performed following manufacturer’s protocol (Abcam ab46074, Cambridge, UK). The absorbance from each well was quantified using a Biotek Synergy H1 Microplate reader (Agilent Technologies, Santa Clara, CA) and the concentration of secreted TRAIL in each sample was determined based on an established standard curve.

### In vitro tumor cell kill studies

CS hydrogels were prepared as previously described with a cell loading density of 5 × 10^6^ to 2 × 10^7^ iNSC/mL of hydrogel (100 μL/mold, n = 6) and incubated at 37°C/5% CO_2_. TRAIL conditioned media was collected as described above. For both U87 and GBM8, 5 × 10^3^ cells were seeded per well in 50 μL standard media and cultured in the plates for about 24 h (day 0). U87 cells were allowed to adhere to the plates, and GBM8 are non-adherent suspension cells line so cells were cultured for 24 h for consistency. On day 1, 100 μL conditioned ReNcell media from either 24 h or 72 h timepoint was added to each well. After an additional 24 h, 150 μL of 10% luciferin PBS solution was added to each well for a final volume of 300 μL. After 15 min of incubation, BLI images were captured using a Spectral Instruments AMI instrument with an exposure of 5 s. U87 or GBM8 cells cultured in standard media, DMEM or EF media respectively, served as controls to normalize BLI signals.

### Statistical analysis

GraphPad Prism Software v 10.5.0 was used to conduct a two-way ANOVA with Tukey’s multiple comparisons test, significance threshold: p < 0.05. Data was presented as mean ± SD.

## Results and Discussion

### In vitro coculture assay

Neural stem cells have shown promise in preclinical studies as a therapeutic strategy for brain tumors, acting as engineered cellular carriers to deliver anticancer molecules that inhibit tumor progression [14]. Our laboratory has previously demonstrated the efficacy of iNSCs as TRAIL-secreting vehicles for targeted cancer therapy [12, 17]. Since then, we have developed second-generation iNSCs (hiNeuroS) with enhanced proliferation, migratory capacity, and improved persistence, which demonstrated superior therapeutic efficacy to first-generation iNSCs (hiNSC) in breast cancer brain metastasis models [20]. To directly compare therapeutic potential and determine if these advantages extend to GBM, both generations of iNSCs were co-cultured with luciferase-expressing U87 and patient-derived GBM8 cells across a range of tumor-to-iNSC ratios. Prior to coculture, a short recombinant TRAIL dose-response study confirmed that GBM8 cells were substantially more sensitive to TRAIL than U87, with an estimated half maximal inhibitory concentration (IC50) of 25–50 ng/mL for GBM8 and no measurable IC50 for U87 up to 1000 ng/mL (**Supplementary Fig. 1**). Based on this difference in sensitivity, GBM8 cocultures included an additional high tumor burden condition (10:1).

**Figure 1.**
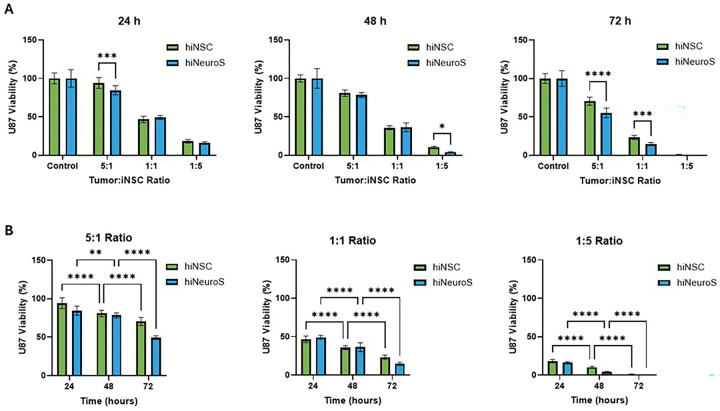
In vitro co-culture assays of iNSCs and U87 cells. Tumor cell viability was measured over 24, 48, and 72 h to compare the cytotoxic efficacy of first- vs second-generation iNSCs. Data is presented as (**A**) time-course comparison and (**B**) effect at varying tumor-to-therapeutic cell ratios. Statistical significance is indicated as: *p < 0.05, **p < 0.01, ***p < 0.001, ****p < 0.0001.

Across both GBM models, second-generation iNSCs consistently induced greater tumor cytotoxicity than the first generation, with a clear time- and dose-dependent relationship. In U87 cocultures, hiNeuroS outperformed hiNSCs at nearly all timepoints and ratios (Fig. 1). At the 5:1 tumor-to-iNSC ratio, hiNeuroS cells reduced viability to 84.5 ± 5.7% at 24 h versus 94.1 ± 6.8% for hiNSCs (p = 0.0009), and this advantage persisted through 72 h (55.2 ± 6.0% vs. 70.4 ± 5.0%, p < 0.0001). At a 1:1 ratio, killing was even more pronounced by 72 h, with hiNeuroS cells reducing viability to 14.6 ± 1.9% compared to 23.2 ± 2.6% (p = 0.0006) with hiNSCs. When iNSCs outnumbered tumor cells (1:5), near-complete elimination was achieved by both generations at 72 h.

**Figure 2.**
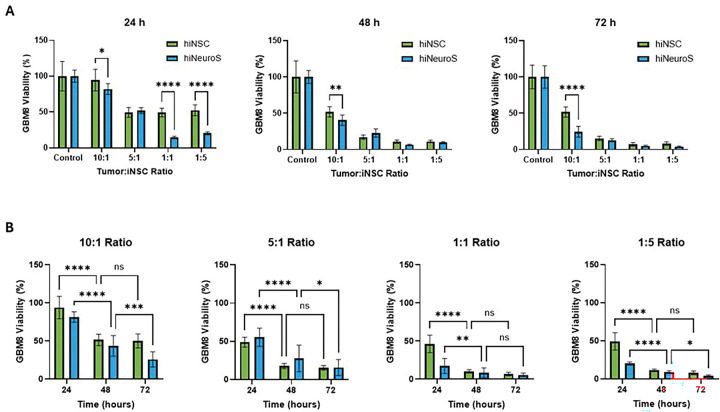
In vitro co-culture assays of iNSCs and GBM8 cells. Tumor cell viability was measured over 24, 48, and 72 h to compare the cytotoxic efficacy of first- vs second-generation iNSCs. Data is presented as (**A**) time-course comparison and (**B**) effect at varying tumor-to-therapeutic cell ratios. Statistical significance is indicated as: *p < 0.05, **p< 0.01, ***p < 0.001, ****p< 0.0001.

The enhanced performance of these next-generation cells was even more apparent in patient-derived GBM8 cells, which demonstrated greater TRAIL sensitivity. As seen in Fig. 2, at the highest 10:1 tumor-to-iNSC ratio, hiNeuroS reduced GBM8 viability to 82.0 ± 7.4% at 24 h compared to 94.5 ± 14.4% for hiNSCs (p = 0.011), and by 72 h this gap widened substantially (24.5 ± 7.1% vs. 51.7 ± 6.7%, with hiNeuroS and hiNSCs respectively, p < 0.0001). At a 1:1 ratio, hiNeuroS elicited a rapid early killing response at 24 h (14.7 ± 1.5%) compared to hiNSCs (49.3 ± 5.7%, p < 0.0001). Again, under the therapeutically favorable 1:5 condition, there was near-zero BLI signal from tumor cells seen by 72 h with both iNSC generations.

The efficacy of hiNeuroS can be attributed to the improved cellular properties previously characterized [20]. Enhanced proliferation enables rapid expansion of therapeutic cell numbers over the 72-hour co-culture period, progressively increasing the cumulative TRAIL exposure. Improved persistence of these neurospheres supports sustained TRAIL secretion and prolonged effect on tumor cells, translating into the time-dependent enhancement of cytotoxicity observed between 24 and 72 hours. Overall, hiNeuroS demonstrated more rapid onset of killing, with significant cytotoxicity evident as early as 24 hours, which is critical for aggressive, rapidly proliferating GBM. In addition, greater efficacy was observed at tumor-dominant ratios (5:1 for U87, 10:1 for GBM8), which is advantageous in situations where GBM cells may outnumber therapeutic iNSCs within the resection cavity. Together, these results confirm that the functional improvements previously described for hiNeuroS in metastatic breast cancer models extend to primary GBM. Their superior potency across multiple ratios, timepoints, and GBM subtypes establishes this next-generation cell line as a distinctly more effective therapeutic payload for scaffold-mediated delivery.

### Preparation of hydrogel formulation

**Figure 3.**
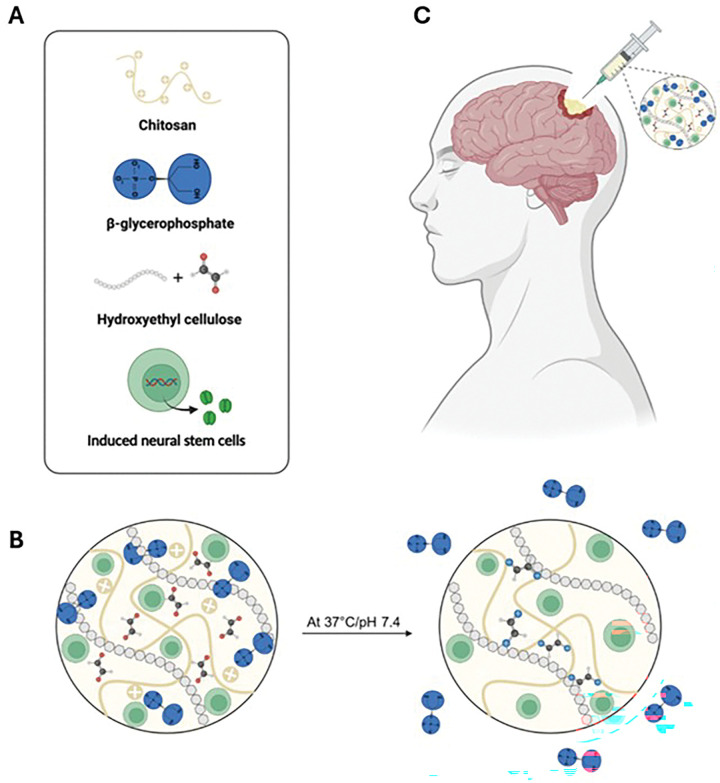
Schematic illustration of injectable hydrogel system. (**A**) Main components of the injectable hydrogel system. (**B**) Crosslinking mechanism that occurs under physiological conditions. (**C**) Injection of the therapy into GBM resection cavity.

Having established the superior anti-GBM efficacy of hiNeuroS, we next characterized their delivery via injectable chitosan hydrogel scaffolds designed to provide sustained local cell retention and overcome the rapid clearance observed with direct injection. The chitosan-based system used was adapted from our prior studies, which demonstrated that CS undergoes a rapid sol-gel transition at physiological temperature and pH through a dual crosslinking mechanism [35–37]. This temperature-dependent transition enables injection of a cell-laden precursor solution that immediately forms a stable gel upon exposure to 37°C, providing high cell retention. β-glycerophosphate (BGP) serves as the primary gelling agent, contributing to the rapid, temperature-dependent sol-gel transition. Hydroxyethyl cellulose (HEC) functionalized with glyoxal provides secondary chemical crosslinking between the glyoxal groups on HEC and the deprotonated amines on the CS backbone via Schiff base reaction [35], reinforcing the network and accelerating gelation kinetic (Fig. 3).

Since the sol-gel transition relies primarily on physical crosslinking with secondary chemical crosslinking occurring under physiological conditions, it is important to assess how encapsulated cells at different densities influence the hydrogel’s physical and chemical properties. To determine the optimal cell density for hydrogel formulations, we investigated gelling kinetics and scaffold properties following cell encapsulation at varying densities. Previous work with this platform using first-generation iNSCs identified 10 million cells/mL as the maximum practical loading density; beyond this point cell clumping and excessive viscosity precluded uniform mixing [37]. We hypothesized that hiNeuroS might tolerate higher-density encapsulation, due to their distinct spheroidal morphology, which would enable delivery of greater therapeutic cell doses. We characterized CS scaffolds loaded with the neurospheres at densities ranging from 5 to 20 million cells/mL.

### Rheological analysis

**Figure 4.**
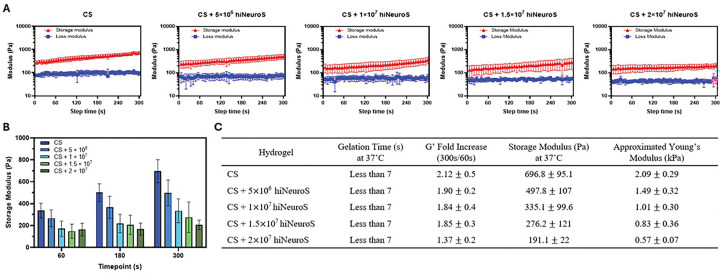
Rheological analysis of injectable hydrogels. (**A**) Gelation time determined by the crossover of storage (G’) and loss (G”) moduli. (**B**) Storage modulus measured at multiple time points during gelation. (**C**) Summary of scaffold mechanical properties at varying iNSC densities; Young’s modulus (E) was calculated as E=2G′(1+ν) assuming ν≈:0.5.

Temperature-dependent rheological analysis demonstrated that all formulations achieved rapid thermoresponsive gelation characteristic of CS hydrogels under physiological conditions (Fig. 4A). The gelation point, defined as the temperature at which storage modulus (G’) crosses over loss modulus (G”), occurred in less than 7 seconds for all formulations. This immediate gelation upon heating to 37°C is important for clinical translation, as it ensures retention of the therapeutic payload upon delivery to the resection cavity.

The evolution of storage modulus during gelation revealed progressive mechanical strengthening of the network (Fig. 4B). From 60 to 300 seconds post-gelation initiation at 37°C, G’ increased more than 2-fold for acellular scaffolds and ranged from 1.37 ± 0.2-fold to 1.90 ± 0.2-fold for cell-laden scaffolds (Fig. 4C). At the 300-second timepoint, a significant density-dependent decrease in storage modulus was observed. 20M cells/mL scaffolds exhibited approximately 73% lower G’ compared to acellular controls, likely reflecting the physical presence of cells disrupting polymer-polymer interactions. Young’s modulus (E) was approximated for each formulation from measured G’ using the following equation:

E=2G′(1+ν)

Assuming a Poisson’s ratio of ν = 0.5 as standard for hydrogels [41, 42], stiffness ranged from 2.09 ± 0.29 kPa for acellular scaffolds to 0.57 ± 0.07 kPa for 20M cells/mL formulations. With this we see values approaching the lower end of native brain tissue stiffness, which is ~ 0.5–1 kPa for gray matter [43, 44]. This softening with increasing cell density may be beneficial, as scaffolds with mechanical properties closer to brain tissue can reduce mechanical stress on surrounding parenchyma during implantation while still supporting iNSC viability and retention. Critically, even at 20M cells/mL where mechanical properties are most reduced, the scaffolds maintained G’ > G” and stable gel formation, indicating sufficient structural integrity for cell encapsulation and delivery. The preservation of rapid gelation kinetics across all cell densities, combined with retained elastic behavior, demonstrates that the CS scaffold can accommodate twice the cell loading of first-generation platforms and demonstrate continued instantaneous gelation.

### Scanning electron microscopy analysis

**Figure 5.**
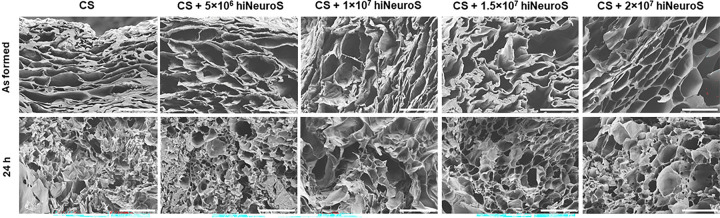
Scaffold microstructure and porosity. Scanning electron microscopy (SEM) images show the cross-section of CS scaffolds as formed and after 24 h incubation in media. Images illustrate scaffold porosity at increasing iNSC concentrations (scale bars, 100 μm; 750 magnification).

The microstructure of hydrogels is important for determining porosity, which is critical for nutrient diffusion, cell survival, and the release of therapeutic proteins. To evaluate scaffold structure, scanning electron microscopy (SEM) imaging was performed on acellular and iNSC-laden CS hydrogels immediately after gelation and following 24 h incubation in complete ReNcell media at 37°C/5% CO_2_. All scaffolds exhibited a highly porous microstructure (Fig. 5), and no noticeable changes in pore size or overall architecture were observed with increasing cell density up to 20M cells/mL. This preservation of porosity suggests that even at the highest cell densities, the scaffold maintains sufficient pathways to support iNSC viability, promote metabolic exchange, and allow for the diffusion of secreted TRAIL into the surrounding microenvironment.

### In vitro mass change study

**Figure 6.**
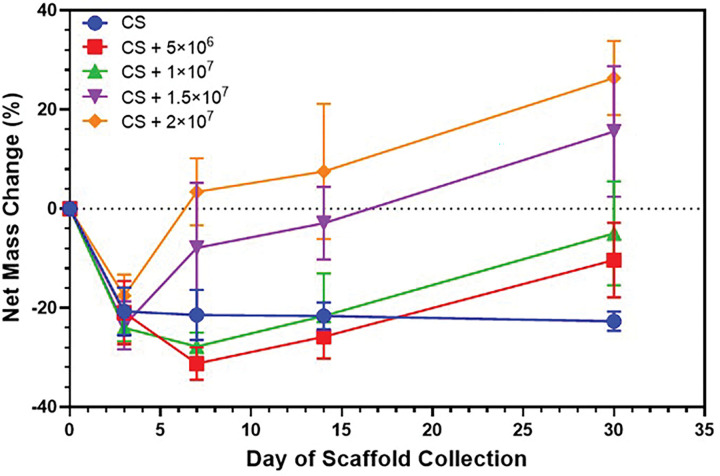
Effect of hiNeuroS on hydrogel mass over time. Percent net mass change of scaffolds measured at collection timepoints relative to initial Day 0 mass (data presented as mean ± SD, n=6). Two-way ANOVA revealed significant main effects of cell density and time, as well as a significant interaction between the two factors (p < 0.0001).

Long-term stability of the hydrogel scaffold is a critical determinant of both therapeutic cell persistence and prolonged TRAIL release within the tumor microenvironment. To evaluate whether the chitosan hydrogel maintains structural integrity during extended culture durations, we performed quantitative dry mass analysis over a 30-day period at cell densities ranging from 0 to 20 million cells/mL. Analysis revealed distinct temporal patterns of net mass change and scaffold stability across different culture conditions (Fig. 6).

The acellular CS control exhibited minimal changes in mass throughout the study (Day 3: −20.7 ± 4.8%; Day 30: −22.7 ± 2.0%), establishing a baseline for scaffold mass in the absence of cellular activity. This behavior is consistent with the slow hydrolytic erosion characteristic of high-molecular-weight chitosan under physiological conditions, and with the expectation that chitosan degradation occurs primarily through enzyme-mediated pathways rather than rapid hydrolysis [45, 46]. We hypothesized that increasing cell density would accelerate scaffold degradation through enhanced secretion of degradative enzymes; however, we observed minimal evidence of cell density-dependent mass loss or degradation.

For all cell-laden hydrogels, Day 3 mass loss mirrored acellular controls, indicating that early mass changes reflect scaffold-associated rather than cell-mediated processes. At Day 7, a clear divergence emerged where lower-density scaffolds elicited mass loss (5M cells/mL: −31.3 ± 3.3%, 10M cells/mL: −27.8 ± 2.8%), whereas higher-density scaffolds (15M cells/mL: −7.9 ± 13.1%, 20M cells/mL: +3.38 ± 6.8%) showed substantially attenuated loss, with 20M approaching neutral net mass change. The divergence became more pronounced by Day 14 with 20M exhibiting net mass gain (+ 7.5 ± 13.7%), whereas 5M and 10M remained in the mass-loss range (− 25.8 ± 4.4% and − 21.6 ± 8.6%, respectively), showing patterns comparable to the acellular control (p > 0.90).

Critically, all cell-laden scaffolds demonstrated significant increase in total mass between Day 14 and Day 30. 20M reached ~ 26% net mass gain relative to Day 0. 10M elicited a net increase from − 21.6 ± 8.6% to − 5.0 ± 10.5% (p < 0.0001), while 5M elicited a mass increase from − 25.8 ± 4.4% to − 10.4 ± 7.5% (p = 0.0038), indicating that even lower-density scaffolds showed overall mass stabilization. The transition from early mass loss to increasing mass retention at all cell seeding densities indicates that cellular activity modulates the overall mass of the scaffold. Although determining precise mechanisms requires additional analyses, potential contributing factors to the observed increase in net mass overtime, particularly with high cell densities include cell proliferation, deposition of extracellular matrix proteins, or cell-mediated changes in scaffold structure. Hydrated mass measurements further supported this interpretation where results showed that 15M and 20M scaffolds retained consistently higher water content than scaffolds seeded at lower densities and acellular controls, indicating density-dependent modulation of scaffold composition or water absorption capacity (**Supplementary Fig. 2**).

Future studies will include incorporating ECM-specific staining and biochemical assays to distinguish polymer degradation from cell- or ECM-derived mass accumulation. Throughout the 30-day study, all scaffolds remained structurally intact. Despite initial mass loss through day 3, SEM imaging confirmed that microstructural features were preserved over time (**Supplementary Fig. 3**), sustaining scaffold integrity and permeability. Cells were able to migrate out of constructs and proliferate on culture surfaces, demonstrating that the hydrogel supports both persistence within the matrix and cell release; properties that are essential for the therapeutic effect of migratory iNSCs. These findings indicate that scaffold stability is maintained throughout the critical early therapeutic window. Overall, our findings reveal no evidence of degradation-driven structural failure over 30 days and support chitosan hydrogels as long-acting, stable platforms for sustained delivery of next-generation iNSC therapeutics.

### Stem cell viability studies

Interestingly, despite dispersing the cells into a single-cell suspension prior to mixing (**Supplementary Fig. 5**), we observed the emergence of multicellular spheroidal aggregates within the scaffold by 72 h. This behavior is characteristic of this hiNeuroS line and may reflect rapid re-establishment of intrinsic cell-cell adhesions within the supportive hydrogel environment. Additionally, confocal imaging revealed that some hiNeuroS migrated out of the hydrogel and adhered to the surrounding culture plate as soon as 24 h, suggesting active motility by the cells. Collectively, these findings demonstrate that the CS hydrogel provides a supportive environment for high-density encapsulation of hiNeuroS while preserving their intrinsic biological behaviors of survival, aggregation, and migration, which are essential for effective function in GBM applications.

### In vitro TRAIL release and tumor cell kill

**Figure 8.**
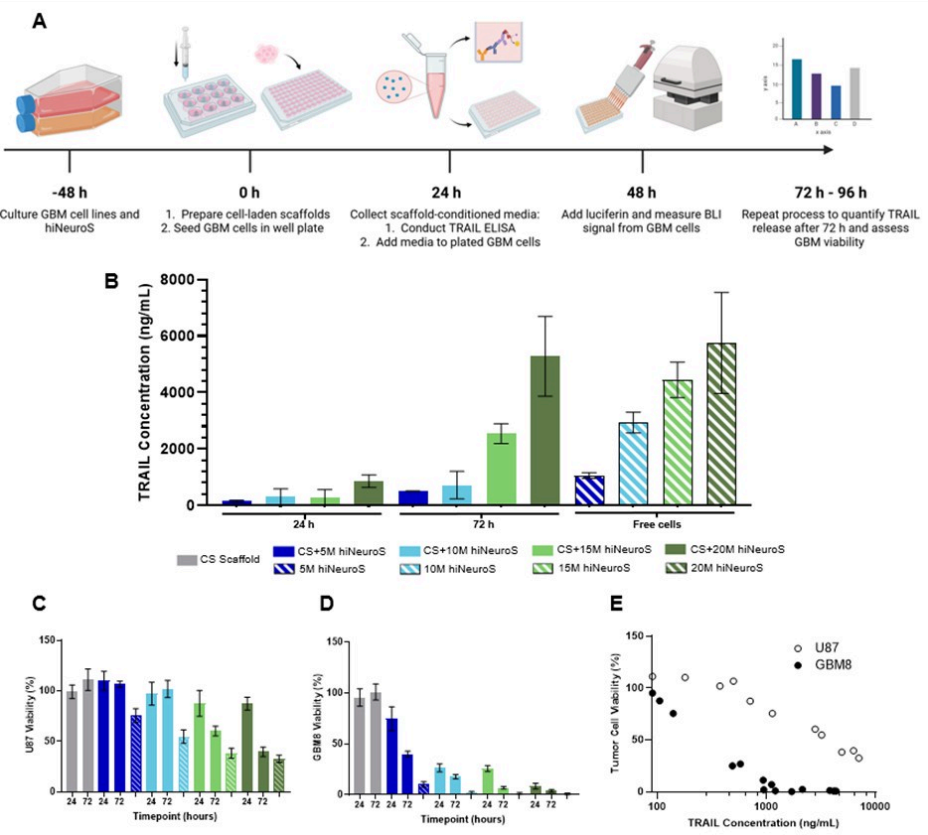
TRAIL release from iNSC-laden scaffolds. (**A**) Schematic of the in vitro TRAIL release assay setup. (**B**) TRAIL concentrations measured by ELISA from scaffolds at 24 and 72 h, compared to 24 h release from free hiNeuroS (control) across varying cell densities. Percent tumor cell viability of (**C**) U87 and (**D**) GBM8 cell lines after exposure to conditioned media, with placebo CS scaffolds as control. (**E**) Correlation between quantified TRAIL concentration in media and observed percent tumor cell viability.

To validate that scaffold encapsulation does not impair the release of our therapeutic protein, we quantified TRAIL release from iNSC-laden scaffolds and assessed the cytotoxicity of the conditioned media against U87 and GBM8 cells. CS hydrogels were prepared with hiNeuroS densities of 5 to 20 million cells/mL and cultured for 24 or 72 hours (Fig. 8A). The resulting conditioned media was collected and TRAIL secretion was quantified using a sandwich-based ELISA, with free (non-encapsulated) iNSCs serving as reference controls. TRAIL protein release was detected from all scaffold conditions, demonstrating that the scaffold does not hinder secretion of the therapeutic protein, as release increased with both time and cell density (Fig. 8B). At 24 hours, TRAIL levels were lower than those produced by free hiNeuroS. This delayed release likely reflects the time required for TRAIL protein to diffuse through the hydrogel matrix network before detection in the conditioned media, similar to observations in prior scaffold-based TRAIL delivery systems [37]. However, by 72 hours, hydrogel formulations seeded with higher densities (15M and 20M cells/mL) exhibited significant increases in TRAIL release from the initial timepoint (p = 0.0041 and p < 0.0001, respectively), reaching concentrations comparable to those obtained with free cells at 24 hours. Notably, the 20M cells/mL scaffold elicited TRAIL concentrations at 72 hours similar to those observed with its respective free-cell control at 24 hours (p = 0.7936), indicating that high-density encapsulation can achieve therapeutically relevant TRAIL concentrations despite the initial physical constraints of the hydrogel environment. In contrast, scaffolds seeded at lower densities (5M and 10M cells/mL) showed no significant difference in TRAIL release between 24 and 72 hours. These results demonstrate that the CS platform supports sustained, cell-dependent TRAIL release, with higher encapsulation densities enabling scaffold-delivered hiNeuroS to produce TRAIL concentrations matching those of free cells.

To evaluate whether scaffold-mediated TRAIL release preserved functional cytotoxicity, GBM cells were exposed to scaffold-conditioned media for 24 h and viability was quantified by bioluminescence imaging (BLI), with acellular CS scaffolds serving as negative controls. GBM cell viability decreased with increasing hiNeuroS concentrations, confirming that TRAIL secreted from encapsulated cells remained biologically active (Fig. 8C–D). Interestingly, conditioned media from acellular CS scaffolds resulted in slightly increased tumor cell viability relative to standard culture controls (U87: p = 0.007; GBM8: p = 0.0251). This minor trophic effect could be attributed to chitosan degradation byproducts or soluble oligosaccharides, which are known to influence proliferation in some cell types, emphasizing that the scaffold itself is not inherently therapeutic and may even be modestly supportive of tumor growth. Despite this tumor-permissive baseline, scaffold conditioned media induced significant cytotoxicity with dose- and time-dependent reductions in glioma cell viability. GBM8 displayed greater sensitivity to TRAIL than U87, which aligns with the known resistance profile of immortalized U87 cells relative to patient-derived models [47, 48]. For U87, cytotoxicity increased with TRAIL concentration but showed no significant difference between 24 and 72 hours for scaffolds seeded with lower cell densities (5M, 10M), consistent with U87’s modestly increased proliferation in CS-conditioned media. In contrast, GBM8 exhibited a significant reduction in viability between 24 and 72 hours at the same densities, reflecting a stronger TRAIL-mediated response.

For U87 cells, 72 h conditioned media reduced viability in a density-dependent manner: 106.5 ± 3.0% (5M), 101.8 ± 8.2% (10M), 60.3 ± 4.6% (15M), and 39.5 ± 4.5% (20M). Scaffolds seeded with the highest cell density elicited 64.4% reduction in tumor cell viability compared to acellular controls (p < 0.0001), representing substantial cytotoxicity despite U87’s relative TRAIL resistance. The temporal enhancement from 24 h to 72 h was highly significant for 15M and 20M scaffolds (p < 0.0001), paralleling increased TRAIL concentrations. GBM8 cells, however, exhibited substantially lower viability across all conditions. Results showed that 24 h conditioned media from 5M scaffolds, the lowest cell density, reduced viability to 74.6 ± 11.3% (p < 0.0001 vs controls). By 72 h, all densities achieved substantial cell kill at 39.7 ± 2.8% (5M), 17.7 ± 2.1% (10M), 6.7 ± 1.2% (15M), and 3.6 ± 1.2% (20M). The 20M scaffold elicited near-complete cell kill (96.4%) compared to controls. Temporal progression was significant across all densities (p < 0.0001 for 5M, 10M, 15M; p = 0.0127 for 20M).

Tumor cell viability was correlated with its corresponding measured TRAIL concentration from the same conditioned media sample, revealing strong inverse relationships for both cell lines (Fig. 8E). GBM8 exhibited a stronger dose-response consistent with enhanced TRAIL sensitivity observed in both our initial TRAIL dose response and direct co-culture. The consistency between conditioned media and co-culture results confirms that TRAIL mediates cytotoxicity in both contexts and that scaffold encapsulation does not compromise TRAIL diffusion. These results demonstrate that hiNeuroS maintain robust TRAIL secretion within the CS scaffold and that secreted TRAIL diffuses through the hydrogel matrix while retaining its bioactivity. The substantial cytotoxicity achieved via paracrine delivery (60% U87 cell kill, 96% GBM8 cell kill with 20M at 72 h) despite a tumor-supportive scaffold environment validates therapeutic efficacy without requiring direct cell-tumor contact. The density-dependent response enables rational dose optimization, with 15–20M seeding densities that are achievable with second-generation iNSCs but not first-generation and can help provide enhanced therapeutic benefit, which will be investigated in future studies. Combined with superior anti-GBM efficacy in coculture studies and favorable scaffold properties, these findings comprehensively validate the CS scaffold/hiNeuroS platform for localized GBM therapy.

## Conclusions

Herein, we evaluated an injectable thermo-responsive chitosan scaffold for the localized delivery of second-generation TRAIL-secreting induced neural stem cells (hiNeuroS) for glioblastoma therapy. hiNeuroS demonstrated enhanced cytotoxic potency over first-generation hiNSCs against both U87 and patient-derived GBM8 cells, achieving faster and more extensive tumor cell kill across multiple effector-to-target ratios. The chitosan hydrogel supported high cell-loading densities (up to 2 × 10^7^ cells/mL) while maintaining > 90% viability, preserving scaffold microarchitecture, and enabling immediate gelation at physiological conditions. Gravimetric mass-loss analysis confirmed that the chitosan matrix remained structurally stable over 30 days, indicating minimal degradation and cell density-dependent mass accumulation. Sustained TRAIL release from cell-laden scaffolds produced therapeutic concentrations comparable to free cells and resulted in dose- and time-dependent tumor cell kill, in patient derived GBM8 cells as well as partially TRAIL-resistant U87 cells. These findings establish that hiNeuroS retain functional activity within the scaffold and that the chitosan matrix provides a stable, biodegradable scaffold for sustaining local cell persistence. While the current work utilized high molecular weight chitosan, future studies could explore lower molecular weight formulations to reduce solution viscosity, fine-tune hydrogel degradation and therapeutic cell release, and potentially increase maximal cell-loading densities. Collectively, this work supports the continued development of injectable thermogelling hydrogel systems to enhance iNSC-based therapies for GBM. Future studies will evaluate in vivo safety, persistence, therapeutic efficacy, and post-implantation phenotypic fate, as the impact of scaffold properties on iNSC gene expression, survival, and differentiation remains unexplored. Overall, this study provides key insights into the design of long-acting biomaterial platforms to advance cell-based therapies for malignant brain tumors.

## Supplementary Material

Supplementary Files

This is a list of supplementary files associated with this preprint. Click to download.

• floatimage5.png

• floatimage1.png

• SupplementaryFigures.docx

## Figures and Tables

**Figure 7.**
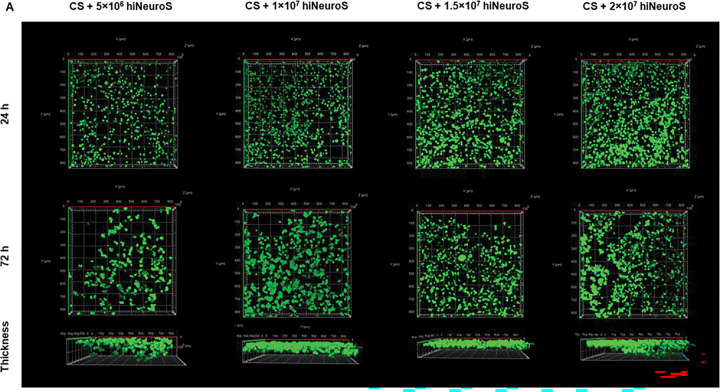
In vitro stem cell viability in CS hydrogels. Z-stack confocal images of scaffolds seeded at various hiNeuroS densities after 24 and 72 h incubation. Live cells are stained with Calcein-AM (green), and dead cells with Ethidium Homodimer-1 (red).

**Table 1.** Hydrogel formulations with varying cell seeding densities

Formulation	CS (% w/v)	BGP (mM)	HEC (mg/mL)	hiNeuroS (per mL hydrogel)

1	2	100	0.5	0
2	2	100	0.5	5 × 10^6^
3	2	100	0.5	1 × 10^7^
4	2	100	0.5	1.5 × 10^7^
5	2	100	0.5	2 × 10^7^

## Data Availability

The data presented in this study are available within the article and Supplementary Material or upon request from the corresponding author.
